# EVIDENCE-BASED INTERGENERATIONAL PRACTICES SUPPORT AFU PRINCIPLES

**DOI:** 10.1093/geroni/igac059.447

**Published:** 2022-12-20

**Authors:** Shannon Jarrott, Jill Juris, Rachel Scrivano

**Affiliations:** The Ohio State University, Columbus, Ohio, United States; Appalachian State University, Boone, North Carolina, United States; The Ohio State University, Columbus, Ohio, United States

## Abstract

Theory and research informed a framework of best intergenerational practice that has proven effective in the field of community programs. Reflecting a number of AFU principles, this set of proven practices can promote positive intergenerational contact in college classrooms as well. Here, we present evidence-based and promising practices salient to the AFU, offering examples from the classroom and suggesting low- and high-tech solutions. For example, the intergenerational practices of offering meaningful roles and engaging youth and older adults in a novel activity can support AFU principle 4 reciprocal sharing of expertise between learners of all ages. Stakeholders, including instructors, university students, older adult learners, and community partners, benefit from preparation for the distinctive needs and opportunities of intergenerational learning experiences in higher education. Effective implementation of intergenerational strategies helps both younger and older learners to reimagine aging, offering the potential to address a critical need of individuals and communities.

